# Paediatric Genetic Diseases: Models, Mechanisms and Therapies

**DOI:** 10.1242/dmm.053177

**Published:** 2026-09-08

**Authors:** Kirsty Hooper

**Affiliations:** The Company of Biologists, Bidder Building, Station Road, Histon, Cambridge CB24 9LF, UK

## Abstract

**Summary:** Disease Models & Mechanisms has launched a Special Issue focused on paediatric genetic disease. Here, we discuss recent research that highlights common themes and challenges in this area of research.

Paediatric diseases – such as cancer, developmental disorders, metabolic disorders and neurodevelopmental disorders – can have strong genetic origins. Despite the expansion of genetic sequencing capabilities, major gaps remain in our understanding of these diseases, leading to limitations in clinical care. Given the unique challenges for biomedical research presented by paediatric genetic diseases, Disease Models & Mechanisms (DMM) has launched a Special Issue, ‘Paediatric Genetic Diseases: Models, Mechanisms and Therapies’ (Box 1), to showcase cutting-edge research and highlight areas of significant progress or prevailing obstacles. This Special Issue is coordinated by DMM Editors James Amatruda (Children's Hospital Los Angeles, Los Angeles, CA, USA) and Elaine Mardis (Nationwide Children's Hospital, Columbus, OH, USA), alongside Guest Editor Ross Cagan (University of Glasgow – Wolfson Wohl Cancer Research Centre, Glasgow, UK). DMM has been a natural home for research in this area over the years, as exemplified below, and we are proud to continue our support for this community of researchers, clinicians, patients, and their families and advocates.

Box 1. DMM Special Issue on Paediatric Genetic Diseases: Models, Mechanisms and Therapies DMM invites you to submit your latest research to our upcoming Special Issue – ‘Paediatric Genetic Diseases: Models, Mechanisms and Therapies’. This issue will be coordinated by DMM Editors James Amatruda (Children's Hospital Los Angeles, Los Angeles, CA, USA) and Elaine Mardis (Nationwide Children's Hospital, Columbus, OH, USA), alongside Guest Editor Ross Cagan (University of Glasgow – Wolfson Wohl Cancer Research Centre, Glasgow, UK). DMM welcomes Research Articles that use a broad range of disease models and approaches to dissect the mechanistic aberrations of paediatric genetic diseases, identify novel drug targets and test emerging therapeutic approaches to, ultimately, foster meaningful clinical progress for patients. We also encourage Resources & Methods submissions that present novel and validated model systems, technical approaches, tools and methodologies that advance our research capabilities for paediatric genetic disease.

The issue will also include specially commissioned Reviews, Perspectives and Interviews from leaders in the field and will be widely promoted online and at key global conferences, guaranteeing maximum exposure for your work. It is scheduled for publication in summer 2027, although note that our continuous publication model means that DMM will publish your article as soon as it is ready; you will not have to wait for the rest of the issue to be finalised. We look forward to receiving your breakthrough paediatric disease research by 7 December 2026.

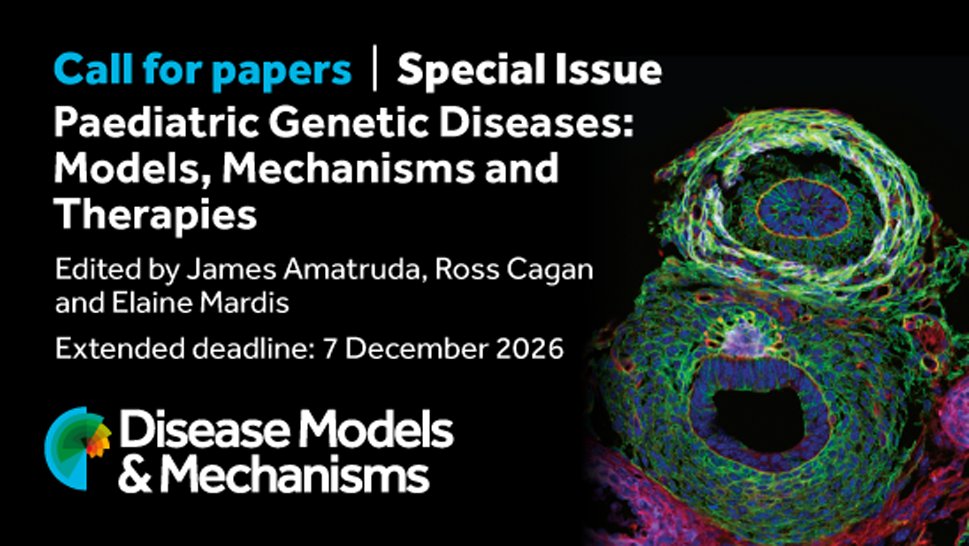

A unifying theme across many studies is the recognition that paediatric disease has strong developmental origins. This is particularly evident in certain childhood cancers. In a Review of fusion-driven paediatric soft-tissue sarcomas, the authors highlighted that the developmental stage in which fusion oncogenes are expressed highly influences tumorigenesis and therapeutic responses in various animal models (Kucinski et al., 2024). The importance of developmental timing is similarly highlighted by van Essen et al. (2024), who generated human induced pluripotent stem cell (iPSC)-derived cerebellar organoids with mutated *PTCH1*, which is important for neuronal development and associated with medulloblastoma. As pathogenic mechanisms of PTCH1 variants differ between mice and humans, this model was important in recapitulating early medulloblastoma initiation. Interestingly, Desingu Rajan et al. (2024) generated an inducible zebrafish model of neurofibromatosis through knockout of the tumour suppressor gene *nf2a/b*, providing a platform to study the effects of neurofibromatosis onset at differing life stages. Together, these studies demonstrate how modelling disease initiation rather than end-stage pathology is becoming a central strategy in paediatric oncology research.

Another important consideration for paediatric cancer is that survivors can experience lifelong adverse effects due to the toxicity of treatments, as emphasised by Jim Amatrdua in his Perspective article (Amatruda, 2021), and by Liz and Jay Scott in an interview about their charity, Alex's Lemonade Stand Foundation (Scott et al., 2026). This is explored further in a study that used a rat model and cultured murine neurons to elucidate the mechanisms by which the chemotherapeutic cisplatin induces monocyte- and macrophage-driven neuroinflammation and sensitisation of pain-sensing neurons (Da Vitoria Lobo et al., 2024). This emphasises a core challenge in paediatric disease, as treatments should aim to have minimal adverse effects on patients' ongoing development and long-term health.

Neurological and neurodevelopmental disorders represent a complex array of diseases, and, owing to their often-rare nature, many of these conditions lack valid and robust model systems. Marshall et al. (2024) established the first mouse model to reproduce key phenotypes of *EEF1A2*-related developmental and epileptic encephalopathies, including problems with movement, decreased growth and signs of seizures. Another study modelled AP2M1-related developmental and epileptic encephalopathies in *Drosophila*, and, although the human phenotype was not recapitulated, it revealed novel insight into neurodevelopmental pathways (Karge et al., 2025). The ultra-rare condition pontocerebellar hypoplasia type 2A was experimentally modelled using patient-derived iPSC lines to generate cerebellar and neocortical organoids that reproduced brain-region-specific growth defects (Kagermeier et al., 2024). In a differing approach, Authier et al. (2024) generated a mouse model of *O*-linked β-*N*-acetylglucosamine transferase intellectual disability that mimicked clinical symptoms and molecular features in the brain. These studies highlight the importance of employing diverse modelling approaches to both recapitulate different aspects of neurological disorders and offer complementary benefits and limitations.

Similar demand for robust model systems is clear for the severe neurodevelopmental syndromes associated with MECP2, with *MECP2* duplication syndrome being associated with overexpression of the protein and Rett syndrome being caused by loss-of-function variants in *MECP2* leading to MECP2 and BDNF insufficiency. Maino et al. (2024) generated a mouse model of *MECP2* duplication syndrome that more faithfully mimicked the genomic landscape and symptoms of the human syndrome. Medeiros et al. (2024) used an established Rett syndrome mouse model to demonstrate that a BDNF-like molecule, LM22A-4, could improve synaptic and behavioural abnormalities. In addition, Morales et al. (2025) provided evidence of neuronal DNA damage in MECP2-deficient neurons from the brains of patients with Rett syndrome and in a rat model, introducing an additional potential disease mechanism. As Rett syndrome is an X-linked disorder, affected brains comprise a ‘mosaic’ of MECP2-sufficent and -deficient neurons, and the differing molecular mechanisms in these neighbouring neurons were examined in both studies (Medeiros et al., 2024; Morales et al., 2025). Collectively, these studies illustrate how precise modelling and interpretation of the unique genetics of these disorders enable better understanding of disease mechanisms and inform therapeutic progress.

Recent developments in neuromuscular disease research are strengthening the translation of mechanistic discoveries. In a Review article, Findlay (2024) outlines the genetic basis of dominantly inherited muscle disorders and discusses promising therapeutic strategies that target their complex disease mechanisms. Furthermore, several studies illustrate the importance of robust preclinical models in this field, such as a new mouse model generated to study Becker muscular dystrophy (Perillat et al., 2025). Canine models can also be used to bridge the translation gap for muscular disorders, as exemplified by a canine model of Duchenne muscular dystrophy that exhibited reduced DWORF expression and can be used in future studies to assess DWORF-targeted gene therapies (Gibson et al., 2025).

A particularly notable theme in neuromuscular disorder research is the ongoing effort to standardise translational pipelines. In a Review-type article, Karuppasamy et al. (2024) addressed a long-standing challenge by proposing standardised zebrafish drug-testing parameters for muscle diseases. Such advances are crucial because therapeutic candidates often fail when experimental approaches differ between laboratories. Reporting negative findings remains equally important to improve efficiency in research (Sansom et al., 2024). Gineste et al. (2025) reported that tamoxifen failed to improve muscle dysfunction in a centronuclear myopathy model, providing valuable evidence that can redirect resources toward more promising interventions. The combined effort to improve modelling and therapeutic development for neuromuscular disorders is building a foundation for tangible clinical outcomes for these childhood disorders.

As muscular disorders often extend to cardiac muscle, cardiomyopathies are closely connected, clinically and mechanistically. Complementary model systems, including mouse models (Fu et al., 2025) and stem cell models (Fullenkamp et al., 2024), are continually advancing to enhance cardiomyopathy research. One study of Alström syndrome highlighted a key consideration when modelling paediatric disorders; Alms1-deficient mice developed features of adult cardiomyopathy but not the infantile presentation (McKay et al., 2024). This observation underscores the ongoing importance of paediatric research, as childhood disease cannot always be inferred from adult disease biology. Distinct developmental contexts may fundamentally alter disease mechanisms and therapeutic responses.

Beyond inherited cardiomyopathies, several studies investigate congenital heart defects and offer another striking illustration of developmental disease mechanisms. Berger et al. (2024) showed that *FBRSL1* is required for heart development and linked *de novo* variants to congenital heart defects in *Xenopus laevis*. Similarly, Berns et al. (2025) identified a homozygous *WNT11* variant associated with congenital heart disease and renal abnormalities in a paediatric patient, and functionally characterised the variant in *Xenopus* embryos, concluding that biallelic *WNT11* dysfunction is linked to syndromal human phenotypes. With advances in genetic sequencing transforming genetic diagnosis of paediatric diseases, model systems, such as *Xenopus*, remain instrumental to fully understanding the functional impact and clinical significance of the genetic variants.

Functional validation studies extend into many distinct paediatric disease areas. Tophkhane et al. (2024) used chicken embryos to validate the pathogenicity of *FZD2* variants associated with Robinow syndrome, while Hampl et al. (2024) revealed craniofacial and neurodevelopmental abnormalities during embryogenesis in a mouse model of the craniofacial disorder known as CDK13-related disorder. These functional validation studies highlight how perturbation of embryonic patterning pathways can produce complex phenotypes.

Sensory disorders provide additional examples of how developmental biology informs disease mechanisms. Wang and Streit (2024) reviewed the developmental mechanisms shared between ear and kidney formation, offering important insights into the cross-talking mechanisms of oto-renal syndromes. Nayak et al. (2025) revealed the mechanistic role of the antioxidant methionine sulfoxide reductase in inner-ear structure formation and hearing function in mice. Pivoting to eye disorders, variants of the *ABCA4* gene are the primary cause of Stargardt disease, which is the leading cause of inherited childhood blindness. A recent study in zebrafish demonstrated that this is due to the role of *ABCA4* in maintaining cone photoreceptor integrity (Willoughby and Jensen, 2025). Together, these studies improve understanding of disease pathogenesis while informing core developmental pathways.

On another note, many paediatric genetic diseases are caused by disrupted metabolic pathways. For instance, Gomez et al. (2025) studied peroxisome biogenesis disorders in *Drosophila* and identified a severity spectrum for patient-derived *PEX2* and *PEX16* variants, refining our understanding of genotype–phenotype relationships in this disease. TANGO2 deficiency disorder is another rare genetic disorder that involves neurodegeneration and metabolic crises. In a Perspective article, Sacher et al. (2024) highlighted lipid imbalance as a major contributor to TANGO2 deficiency disorder and proposed vitamin B5 supplementation as a potential treatment targeting this imbalance. By enriching our understanding of disease mechanisms, the research community is expanding therapeutic possibilities for paediatric diseases.

In this vein, effective gene therapies are fast becoming a reality for some paediatric genetic diseases. Pratt et al. (2025) evaluated gene replacement strategies using a mouse model and a human iPSC model of *MTRFR* deficiency, which causes mitochondrial diseases such as Behr's syndrome and Leigh syndrome. As disease mechanisms become clearer and models become more predictive, the prospect of durable genetic treatments for paediatric diseases becomes increasingly realistic. Yet the challenge of ensuring equitable access to these transformative therapies remains prominent, especially for rare diseases, as discussed in a Perspective article from Fox and Booth (2024).

Owing to the rare nature of many paediatric diseases, patient advocacy organisations are essential in helping to prioritise research efforts to maximise clinical impact. In DMM's ‘The Patient's Voice’ series, we interviewed several advocates for rare disease research who outline the immense and unique challenges that these patients and their families face (Krishnaraj and Rajasimha, 2024; Stedman, 2024). While scientific fields progress, it is important to encourage collaborations that enable patient-led research to thrive.

Overall, it is clear that the converging fields encompassing paediatric genetic disease are transforming alongside rapidly advancing experimental and clinical capabilities. Across the diverse disease groups, common themes emerge in their complex genetic and developmental origins. With model systems becoming more sophisticated and clinically relevant, mechanistic discoveries are becoming increasingly actionable. Fundamentally, these advances are narrowing the gap between gene discovery and patient benefit.

## References

[DMM053177C1] Amatruda, J. F. (2021). Modeling the developmental origins of pediatric cancer to improve patient outcomes. *Dis. Model. Mech.* 14, dmm048930. 10.1242/dmm.048930 PMC7927656 33619212

[DMM053177C2] Authier, F., Ondruskova, N., Ferenbach, A. T., McNeilly, A. D. and van Aalten, D. M. F. (2024). Neurodevelopmental defects in a mouse model of *O*-GlcNAc transferase intellectual disability. *Dis. Model. Mech.* 17, dmm050671. 10.1242/dmm.050671 PMC11095632 38566589

[DMM053177C3] Berger, H., Gerstner, S., Horstmann, M.-F., Pauli, S. and Borchers, A. (2024). Fbrsl1 is required for heart development in *Xenopus laevis* and *de novo* variants in *FBRSL1* can cause human heart defects. *Dis. Model. Mech.* 17, dmm050507. 10.1242/dmm.050507 PMC11128277 38501224

[DMM053177C4] Berns, H., Weber, D., Haas, M., Bakey, Z., Brislinger-Engelhardt, M. M., Schmidts, M. and Walentek, P. (2025). A homozygous human *WNT11* variant is associated with laterality, heart and renal defects. *Dis. Model. Mech.* 18, dmm052211. 10.1242/dmm.052211 PMC12091873 40200693

[DMM053177C5] Da Vitoria Lobo, M., Hardowar, L., Valentine, T., Tomblin, L., Guest, C., Sharma, D., Dickins, B., Paul-Clark, M. and Hulse, R. P. (2024). Early-life cisplatin exposure induces neuroinflammation and chemotherapy-induced neuropathic pain. *Dis. Model. Mech.* 17, dmm052062. 10.1242/dmm.052062 PMC11625889 39428813

[DMM053177C6] Desingu Rajan, A. R., Huang, Y., Stundl, J., Chu, K., Irodi, A., Yang, Z., Applegate, B. E. and Bronner, M. E. (2024). Generation of a zebrafish neurofibromatosis model via inducible knockout of *nf2a/b*. *Dis. Model. Mech.* 17, dmm050862. 10.1242/dmm.050862 PMC11646113 39415595

[DMM053177C7] Findlay, A. R. (2024). Dominantly inherited muscle disorders: understanding their complexity and exploring therapeutic approaches. *Dis. Model. Mech.* 17, dmm050720. 10.1242/dmm.050720 PMC11574355 39501809

[DMM053177C8] Fox, T. A. and Booth, C. (2024). Improving access to gene therapy for rare diseases. *Dis. Model. Mech.* 17, dmm050623. 10.1242/dmm.050623 PMC11051979 38639083

[DMM053177C9] Fu, X., Yuan, W., Li, J., Wan, K., Ge, M., Pan, B. and Lu, T. (2025). Establishment and functional studies of a model of cardiomyopathy with cardiomyocyte-specific conditional knockout of *Arhgef18*. *Dis. Model. Mech.* 18, dmm052172. 10.1242/dmm.052172 PMC11992352 40159883

[DMM053177C10] Fullenkamp, D. E., Willis, A. B., Curtin, J. L., Amaral, A. P., Dittloff, K. T., Harris, S. I., Chychula, I. A., Holgren, C. W., Burridge, P. W., Russell, B. et al. (2024). Physiological stress improves stem cell modeling of dystrophic cardiomyopathy. *Dis. Model. Mech.* 17, dmm050487. 10.1242/dmm.050487 PMC10820750 38050701

[DMM053177C11] Gibson, A. M., Pan, X., Brito-Estrada, O., Teixeira, J. A., Carl, L. K., Yue, Y., Kamradt, M. L., Burke, M. J., Wadding-Lee, C. A., Yao, G. et al. (2025). DWORF expression is reduced in a large animal model of Duchenne muscular dystrophy. *Dis. Model. Mech.* 18, dmm052285. 10.1242/dmm.052285 PMC12233064 40557917

[DMM053177C12] Gineste, C., Reiss, D. and Laporte, J. (2025). Tamoxifen treatment fails to improve muscle dysfunction in a model of recessive *RYR1*-linked centronuclear myopathy. *Dis. Model. Mech.* 18, dmm052462. 10.1242/dmm.052462 PMC12805646 41459639

[DMM053177C13] Gomez, V. A., Kanca, O., Jangam, S. V., Srivastav, S., Andrews, J. C. and Wangler, M. F. (2025). Distinguishing *PEX2* and *PEX16* gene variant severity for mild, severe and atypical peroxisome biogenesis disorders. *Dis. Model. Mech.* 18, dmm052258. 10.1242/dmm.052258 PMC12352285 40621817

[DMM053177C14] Hampl, M., Jandová, N., Lusková, D., Nováková, M., Szotkowská, T., Čada, Š., Procházka, J., Kohoutek, J. and Buchtová, M. (2024). Early embryogenesis in CHDFIDD mouse model reveals facial clefts and altered cranial neurogenesis. *Dis. Model. Mech.* 17, dmm050261. 10.1242/dmm.050261 PMC11212636 38511331

[DMM053177C15] Kagermeier, T., Hauser, S., Sarieva, K., Laugwitz, L., Groeschel, S., Janzarik, W. G., Yentür, Z., Becker, K., Schöls, L., Krägeloh-Mann, I. et al. (2024). Human organoid model of pontocerebellar hypoplasia 2a recapitulates brain region-specific size differences. *Dis. Model. Mech.* 17, dmm050740. 10.1242/dmm.050740 PMC11552497 39034883

[DMM053177C16] Karge, R. A., Fischer, F. P., Schüth, H., Wechner, A., Peter, S., Kilo, L. A., Dichter, M., Voigt, A., Tavosanis, G., van Loo, K. M. J. et al. (2025). Modeling AP2M1 developmental and epileptic encephalopathy in *Drosophila*. *Dis. Model. Mech.* 18, dmm052419. 10.1242/dmm.052419 PMC12673965 41017589

[DMM053177C17] Karuppasamy, M., English, K. G., Henry, C. A., Manzini, M. C., Parant, J. M., Wright, M. A., Ruparelia, A. A., Currie, P. D., Gupta, V. A., Dowling, J. J. et al. (2024). Standardization of zebrafish drug testing parameters for muscle diseases. *Dis. Model. Mech.* 17, dmm050339. 10.1242/dmm.050339 PMC10820820 38235578

[DMM053177C18] Krishnaraj, P. and Rajasimha, H. K. (2024). Cross-border rare disease advocacy: Preethi Krishnaraj interviews Harsha Rajasimha. *Dis. Model. Mech.* 17, dmm050672. 10.1242/dmm.050672 PMC10846506 38270284

[DMM053177C19] Kucinski, J. P., Calderon, D. and Kendall, G. C. (2024). Biological and therapeutic insights from animal modeling of fusion-driven pediatric soft tissue sarcomas. *Dis. Model. Mech.* 17, dmm050704. 10.1242/dmm.050704 PMC11225592 38916046

[DMM053177C20] Maino, E., Scott, O., Rizvi, S. Z., Chan, W. S., Visuvanathan, S., Zablah, Y. B., Li, H., Sengar, A. S., Salter, M. W., Jia, Z. et al. (2024). An *Irak1-Mecp2* tandem duplication mouse model for the study of *MECP2* duplication syndrome. *Dis. Model. Mech.* 17, dmm050528. 10.1242/dmm.050528 PMC11552499 38881329

[DMM053177C21] Marshall, G. F., Fasol, M., Davies, F. C. J., Le Seelleur, M., Fernandez Alvarez, A., Bennett-Ness, C., Gonzalez-Sulser, A. and Abbott, C. M. (2024). Face-valid phenotypes in a mouse model of the most common mutation in *EEF1A2*-related neurodevelopmental disorder. *Dis. Model. Mech.* 17, dmm050501. 10.1242/dmm.050501 PMC10855229 38179821

[DMM053177C22] McKay, E. J., Luijten, I., Broadway-Stringer, S., Thomson, A., Weng, X., Gehmlich, K., Gray, G. A. and Semple, R. K. (2024). Female Alms1-deficient mice develop echocardiographic features of adult but not infantile Alström syndrome cardiomyopathy. *Dis. Model. Mech.* 17, dmm050561. 10.1242/dmm.050561 PMC11225586 38756069

[DMM053177C23] Medeiros, D., Ayala-Baylon, K., Egido-Betancourt, H., Miller, E., Chapleau, C., Robinson, H., Phillips, M. L., Yang, T., Longo, F. M., Li, W. et al. (2024). A small-molecule TrkB ligand improves dendritic spine phenotypes and atypical behaviors in female Rett syndrome mice. *Dis. Model. Mech.* 17, dmm050612. 10.1242/dmm.050612 PMC11139040 38785269

[DMM053177C24] Morales, A., Korsakova, E., Mansooralavi, N., Soliman, P., Jahanbani, S., Olsen, M. L., Badhuri, A. and Lowry, W. E. (2025). Evidence of neuronal DNA damage in the brains of patients with Rett syndrome. *Dis. Model. Mech.* 18, dmm052358. 10.1242/dmm.052358 PMC12452059 40739790

[DMM053177C25] Nayak, G., Richard, E. M., Lee, B. C., Riordan, G. P., Belyantseva, I. A., Manta, B., Friedman, T. B., Gladyshev, V. N. and Riazuddin, S. (2025). MSRB3 antioxidant activity is necessary for inner ear cuticular plate structure and hair bundle integrity. *Dis. Model. Mech.* 18, dmm052194. 10.1242/dmm.052194 PMC12403523 40827380

[DMM053177C26] Perillat, L. O. M., Wong, T. W. Y., Maino, E., Ahmed, A., Scott, O., Hyatt, E., Delgado-Olguin, P., Visuvanathan, S., Ivakine, E. A. and Cohn, R. D. (2025). Generation and characterization of a mouse model of Becker muscular dystrophy with a deletion of *Dmd* exons 52 to 55. *Dis. Model. Mech.* 18, dmm050595. 10.1242/dmm.050595 PMC12519546 39099311

[DMM053177C27] Pratt, S. L., Zarate-Mendez, M., Koludarova, L., Jansson, S., Airavaara, M., Hlushchuk, I., Coleman, D., Heffner, C., Horvath, R., Battersby, B. J. et al. (2025). Evaluating the feasibility of gene replacement strategies to treat *MTRFR* deficiency. *Dis. Model. Mech.* 18, dmm052120. 10.1242/dmm.052120 PMC12171093 40452409

[DMM053177C28] Sacher, M., DeLoriea, J., Mehranfar, M., Casey, C., Naaz, A., MacKenzie, S. J. and Gamberi, C. (2024). TANGO2 deficiency disorder is predominantly caused by a lipid imbalance. *Dis. Model. Mech.* 17, dmm050662. 10.1242/dmm.050662 PMC11179719 38836374

[DMM053177C29] Sansom, O., Bogani, D., Reichenbach, L. and Wells, S. (2024). Negative equity – the value of reporting negative results. *Dis. Model. Mech.* 17, dmm050937. 10.1242/dmm.050937 PMC11381924 39212951

[DMM053177C30] Scott, L., Scott, J. and Alex's Lemonade Stand Foundation. (2026). Alex's Lemonade Stand Foundation squeezes in hope for childhood cancer: an interview with Liz and Jay Scott. *Dis. Model. Mech.* 19, dmm052967. 10.1242/dmm.052967 PMC13225204 42084136

[DMM053177C31] Stedman, I. (2024). Igniting an autoinflammatory disease community: an interview with Ian Stedman. *Dis. Model. Mech.* 17, dmm050642. 10.1242/dmm.050642 PMC10820735 38231149

[DMM053177C32] Tophkhane, S. S., Fu, K., Verheyen, E. M. and Richman, J. M. (2024). Craniofacial studies in chicken embryos confirm the pathogenicity of human *FZD2* variants associated with Robinow syndrome. *Dis. Model. Mech.* 17, dmm050584. 10.1242/dmm.050584 PMC11247504 38967226

[DMM053177C33] van Essen, M. J., Apsley, E. J., Riepsaame, J., Xu, R., Northcott, P. A., Cowley, S. A., Jacob, J. and Becker, E. B. E. (2024). *PTCH1*-mutant human cerebellar organoids exhibit altered neural development and recapitulate early medulloblastoma tumorigenesis. *Dis. Model. Mech.* 17, dmm050323. 10.1242/dmm.050323 PMC10924233 38411252

[DMM053177C34] Wang, S. X. and Streit, A. (2024). Shared features in ear and kidney development – implications for oto-renal syndromes. *Dis. Model. Mech.* 17, dmm050447. 10.1242/dmm.050447 PMC10886756 38353121

[DMM053177C35] Willoughby, J. J. and Jensen, A. M. (2025). Abca4, mutated in Stargardt disease, is required for structural integrity of cone outer segments. *Dis. Model. Mech.* 18, dmm052052. 10.1242/dmm.052052 PMC11744051 39610324

